# Hazardous Traffic Event Detection Using Markov Blanket and Sequential Minimal Optimization (MB-SMO)

**DOI:** 10.3390/s16071084

**Published:** 2016-07-13

**Authors:** Lixin Yan, Yishi Zhang, Yi He, Song Gao, Dunyao Zhu, Bin Ran, Qing Wu

**Affiliations:** 1 Intelligent Transport Systems Research Center, Wuhan University of Technology, Wuhan 430063, China; yanlixinits@126.com (L.Y.); gaosong@whut.edu.cn (S.G.); dunyaozhu@foxmail.com (D.Z.); wq@whut.edu.cn (Q.W.); 2 Engineering Research Center for Transportation Safety, Ministry of Education, Wuhan 430063, China; 3 Department of Civil and Environmental Engineering, University of Wisconsin-Madison, Madison, WI 53706, USA; bran@engr.wisc.edu; 4 Management School, Jinan University, Guangzhou 510632, China; easezh@126.com

**Keywords:** hazardous traffic event, Markov blanket, sequential minimal optimization, naturalistic driving, traffic safety

## Abstract

The ability to identify hazardous traffic events is already considered as one of the most effective solutions for reducing the occurrence of crashes. Only certain particular hazardous traffic events have been studied in previous studies, which were mainly based on dedicated video stream data and GPS data. The objective of this study is twofold: (1) the Markov blanket (MB) algorithm is employed to extract the main factors associated with hazardous traffic events; (2) a model is developed to identify hazardous traffic event using driving characteristics, vehicle trajectory, and vehicle position data. Twenty-two licensed drivers were recruited to carry out a natural driving experiment in Wuhan, China, and multi-sensor information data were collected for different types of traffic events. The results indicated that a vehicle’s speed, the standard deviation of speed, the standard deviation of skin conductance, the standard deviation of brake pressure, turn signal, the acceleration of steering, the standard deviation of acceleration, and the acceleration in Z (G) have significant influences on hazardous traffic events. The sequential minimal optimization (SMO) algorithm was adopted to build the identification model, and the accuracy of prediction was higher than 86%. Moreover, compared with other detection algorithms, the MB-SMO algorithm was ranked best in terms of the prediction accuracy. The conclusions can provide reference evidence for the development of dangerous situation warning products and the design of intelligent vehicles.

## 1. Introduction

Road traffic crashes have become the key cause of death of the population in the current years. In China, although crashes have decreased in recent years (about approximately 60% reduction from 2000 to 2012) and roadway fatalities have decreased from 93,853 to 62,387 during these years [1], the total number of crashes and fatalities are still at high levels [2]. A survey by the U.S. National Highway Traffic Safety Administration (NHTASA) showed that 60% of crashes could be avoided if drivers could receive notice 0.5 s in advance [3]. However, the traffic system is one of the most complex and important components in modern society. The risk factors from humans, vehicles, roads and the environment all contribute to the occurrence of hazardous traffic events. Meanwhile, during driving and due to a lack of experience or other reasons, drivers can easily become nervous or make mistakes during vehicle operation, which can lead to crashes. These crashes could be avoided if a warning system or automatic driving system could identify dangerous situations and warn or replace drivers to finish the driving task when hazardous traffic events appeared [4,5]. Thus, a methodology that could reduce the crash rate is necessary. The objective of this study is to extract the risk factors which significantly influenced the occurrence of hazardous traffic events, and we also tried to establish a detection model for identifying different types of traffic events.

Hazardous traffic events are not only difficult to define, but also uncontrollable and uncertain. Dingus et al. [6] defined three types of traffic events as follows:

- Crash: situations in which there is physical contact between the subject vehicle and another vehicle, fixed object, pedestrian, cyclist or animal.
- Near-Crash: situations requiring a rapid, severe, evasive maneuver to avoid a crash.
- Incident: situations requiring an evasive maneuver of less magnitude than for a near-crash.

Considering the safety of an on-road experiment and the difficulty of crash data collection in China, crash and near-crash events (events which appear in China’s transportation industry standard JTT 916-2014 [7]) are defined as hazardous events, and the most common hazardous events consist of pedestrians crossing the road, formal vehicle emergency braking, and so on. Risky traffic events could be defined as those in which the driver does not need to take emergency action, but only require the driver’s attention to avoid crashes. Safe traffic event could be defined as situations which do not influence driver’s normal driving behavior, and the occurrence of those traffic events does not make the driver feel nervous or fearful [8].

To identify hazardous or risky traffic events during normal driving, it is critical to extract the impact factors that have significant influences on risky driving and explore the characteristics of different types of traffic events. The following paragraphs provide a brief review of related studies regarding impact factors of hazardous traffic events, as well as related research on methods to distinguish the different types of traffic events.

The appearance of traffic events could be affected by many factors, e.g., drivers, vehicles, the road and the environment. Bauer et al. [9] found that road curvature has a strong correlation with the traffic incidents and easily leads to the occurrence of crashes. Greibe et al. [10] studied the relationship between road links (such as parings, crossings) and crashes. Kamijo et al. [11] suggested that the number of traffic lanes, intersections and traffic lights could lead to more crashes and risky driving. Deoña et al. [12] tried to use the factors of speed limits, safety distance, overtaking rules and drivers’ distractions to identify the crash risk during the driving. Estimation of hazardous traffic events based on a driver’s personality traits and other relevant variables has also been standard practice in actuarial research; previous studies have also found that crash risk was impacted by a driver’s experience. An inexperienced driver is less likely to scan the roadway than experienced drivers are [13,14]. Efforts have been carried out to deeply analyze the vehicles’ state factors, such as speed, brake, and acceleration, which affect crashes and hazardous traffic events [15]. Moreover, the environment, which includes the weather, traffic flow, and visibility, also has a great influence on the occurrence of crashes [16].

All in all, due to the complexities of the traffic environment, there are many factors that could influence the occurrence of crashes. It is necessary to explore an effective method for extracting key features to classify the different types of traffic events. Many feature selection methods have been used in the area of traffic safety. Montella et al. [17] focused on the crashes of pedestrians and powered two-wheel vehicles. The decision tree (DT) and association rule model were used to identify the significant impact factors of crashes. For pedestrians, the results showed that the road type, lighting conditions, vehicle type, and pedestrian age are the main factors associated with crashes. For powered two-wheel vehicles, they concluded that rainy weather, run-off-the-road, the cure of the road, and the capacity of the vehicle is the key factors that influence the fatal severity. Bing et al. [18] used the principal components analysis (PCA) method to detect hazardous traffic incidents, and three main components were extracted based on the eleven initial factors. Many other feature selection methods such as feature ranking [19], linear forward selection [20], and subset size forward selection [21] have been used to extract key features.

With the development of computer science and sensor technology, the methodology of hazardous traffic event detection has multiplied [22]. Currently, the traffic event detection methods are divided into the three main types (including data analysis, observation method, and expert experiences).

Data analysis is a popular and mainstream method for detecting different types of crashes. Vehicle-based signals, such as a vehicle’s speed, the depth of the brake pedal, the acceleration, the steering wheel movement, current position, and lane position has been used to identify dangerous scenes [23]. For example, Hu et al. [24] investigated traffic accident scenario hotspots based on GPS data. As another example, Florez et al. [25] used a video stream to classify dangerous scenes based on the characteristics of a vehicle’s trajectory. Similarly, behavioral and environment characteristics, such as a driver’s experience, sex, physiological state, eye closure rate, and weather, have been collected and analyzed to identify dangerous traffic events. A previous study [26] found that the sudden movement of a driver’s torso may indicate the occurrence of a traffic incident. Donmez et al. [27] reported that a driver’s off-road glances behavior may easily cause the occurrence of dangerous traffic event. Moreover, the observation method and expert experiences have been widely used to evaluate the risk level of traffic incidents. The key steps of the field observation method are that many observers are employed to add to the record of critical-incident events; these methods always result in a waste of human resources and economic resources. As a simple and easy operating method to identify traffic events, expert analysis only focuses on a video or image of a traffic event, and through the experts’ experience, the risk level of selected traffic scenes is ensured. However, because of inconsistencies resulting from using different experts, it is always difficult to distinguish hazardous traffic events accurately when the traffic environment is complex [28]; moreover, it is not suitable to predict hazardous traffic events in real-time using this method.

In recent years, intelligent computing technology has developed rapidly, and as a typical data analysis method, machine learning algorithms have been used to address multi-sensor information to predict the driving risk. For example, Hassan et al. [29] applied artificial neural network models to predict the crash risk at a signalized intersection. The results showed that the multilayer perceptron (MLP) neural network model has a better generalization performance when predicting the risk at the intersection. Jiao et al. [30] used fault tree analysis (FTA) to develop a dangerous situation prediction system. Further, Wakita et al. [31] proposed a nonparametric method via driving behavior signals to identify driving risk. The results showed that an identification rate of 73% could be reached. Moreover, many existing studies have used classification models to deeply analyze or identify the occurrence of crashes. Sohn and Shin [32] used the accuracy measure to evaluate the accuracy of a neural network, logistic regression, and decision table. The results showed that the three models exhibited a similar level of identification. Other classification algorithms, such as the decision tree (ID3), Classification and Regression Tree (CART), Radial basis function network (RBFNETWORK), and Naïve Bayes (NB), have also been used to identify risky traffic incidents or crashes [33].

Thus far, little research has been found regarding the study of the identification of hazardous traffic events during the real-world driving process, especially in the context of China. The relationship between influence factors from driver-vehicle-road-environment and traffic hazards has already been explored in a previous study. However, little research has focused on the feature selection method of hazardous traffic events. In addition, the hazardous traffic event detection algorithm is an important aspect that still requires improvement. Thus, we conducted a series of on-road experiment to collect the data of multi-sensors while the hazardous traffic event occurred, and the relationship between the impact factors and different types of traffic events was also analyzed. In addition, one of the key objectives of this study is to explore an effective method for evaluating the safety of traffic incidents in the mixed traffic of a Chinese metropolitan area-, i.e., Wuhan.

**Figure 1:**
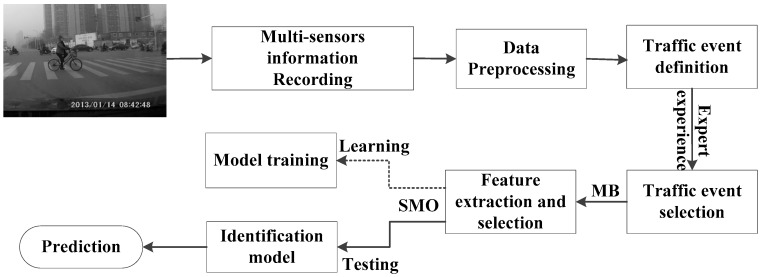
The proposed system for predicting hazardous traffic events.

The technical flowchart of this paper is shown in Figure 1. First, the experimental design and the data collection, including the participants, tasks, instruments, types of data and the origin data preprocessing method, are introduced. Then, a new feature selection algorithm called the Markov Blanket (MB) is introduced to find the key factors that have strong correlation with the different types of traffic incidents. Third, the sequential minimal optimization (SMO) algorithm is selected to establish a hazardous traffic event identification model. Finally, the accuracy of the classification model is evaluated by using a receiver operating characteristic curve (ROC) and many other statistical indexes.

## 2. Methods

### 2.1. Markov Blanket for Evaluating Risk Factors

In this study, we introduce the Markov blanket theory into the area of feature selection with respect to hazardous traffic events. Several researchers have suggested, intuitively, that the Markov blanket of a dependent variable (or target) *T* should be classified as a key concept in solving the variable selection problem. In the context of traffic safety, the Markov blanket of the target T, i.e., risk or the concepts related to safety, is the best group of key factors that have a significant influence on *T* [20,34,35,36].

The Markov blanket of the dependent variable *T*, denoted as MB(T), is a minimal set of variables (or factors, features; hereafter, we use these terms interchangeably) conditioned on which all other variables are probabilistically independent of T (Definition 1). Thus, knowing the values of MB(*T*) is sufficient to determine the probability distribution of *T*, and the values of all other attributes become superfluous [35,36]. Obviously, we can only use attributes in MB(*T*) instead of all the attributes for optimal prediction. Moreover, under certain conditions (faithfulness to a Bayesian network), MB(*T*) is the subset that contains the parents, the children and the parents of the children of the target T in the Bayesian network [37].

**Definition 1 (Markov blanket) [37].**  *The Markov blanket of a target attribute T ∈ V, denoted as MB(T), is a minimal subset of attributes for which:*

(T⊥V−MB(T)−T|MB(T)) and T∉MB(T)

*where V is the set of all attributes in the domain. Symbol ‘*⊥*’ denotes independence. ‘-’ denotes set minus, and ‘|’ means ‘conditioning on’*.

**Figure 2:**
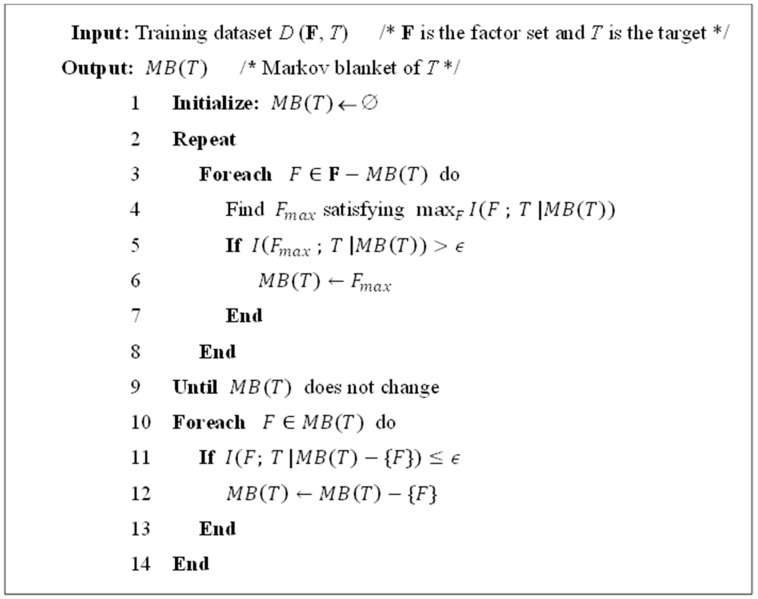
IAMB algorithm.

Given the faithfulness assumption, the probability distribution of *T* is completely determined by the values of variables in MB(*T*). Thus, the Markov blanket is widely applied for the feature selection problem, and a basic algorithm, which called the Incremental Association Markov Blanket (IAMB) algorithm [36], is selected to discover the Markov blanket. The algorithm is described in Figure 2.

As shown in Figure 2, the IAMB algorithm contains two phases, growing phase (lines 2–9) and shrinking phase (lines 10–14). The growing phase of IAMB guarantees finding a Markov boundary of the target *T*, i.e., all of the other factors will be redundant to *T* given such a boundary. The shrinking phase of IAMB tries to further eliminate the redundancy in the Markov boundary discovered in the prior phase. Note that the conditional independence test in IAMB is controlled by the preselected threshold ϵ, which will be nominally set to 0.01, as suggested in [37]. Note that I(∙|∙) in the IAMB algorithm represents the conditional mutual information, where the size of the conditioning set in I(∙|∙) will change at each iteration. In this paper, the conditional mutual information will be implemented according to [20].

### 2.2. The Sequential Minimal Optimization for Detecting Hazardous Traffic Events

After ensuring the factors that contribute to hazardous traffic events based on the Markov blanket algorithm, a detection model was developed to classify the different types of traffic events based on the sequential minimal optimization (SMO) algorithm; the SMO as an improved SVM algorithm, which is used to solve a large-scale Quadratic Programming (QP) problem, was put forward by Platt, in this case, the QP problem could be described as Equations (1) and (2), and the mathematical expression for this algorithm in this study is similar with the article published by Platt in 1998 [38]. The core of SMO algorithm is to decompose the overall QP problem into numerical routine for several sub-problems. The QP problem is solved only if the point in Equations (1) and (2) fulfilled the Karush-Kuhn-Tucker (KKT) conditions shown in Equation (3) and $Q_{ij}=y_{i}y_{j}k\left(X_{i},X_{j}\right)$ is positive semi-definite:

(1) $$\text{Maximize}:\;R\left(\alpha_{i}\right)=\overset{l}{\underset{i=1}{\sum }}\alpha_{i}-\overset{l}{\underset{i=1}{\sum }}\overset{l}{\underset{j=1}{\sum }}\alpha_{i}\alpha_{j}y_{i}y_{j}k\left(X_{i},X_{j}\right)$$

(2) $$\text{Subject to}:\;\overset{l}{\underset{i=1}{\sum }}\alpha_{i}y_{i}=0;\;0\leq \alpha_{i}\leq c,\;i=1,\ldots ,l$$

KKT conditions:

(3) $$\{\begin{array}{c} \alpha_{i}=0=>y_{i}f\left(X_{i}\right)\geq 1\\ 0<\alpha_{i}<c=>y_{i}f\left(X_{i}\right)=1\\ \alpha_{i}=c=>y_{i}f\left(X_{i}\right)\leq 1 \end{array}$$

where $k\left(X_{i},X_{j}\right)$ is the kernel function; $\alpha_{i}$ is the Lagrange multiplier to be optimized; c is the regularization constant predetermined by users. The KKT conditions can give an evaluation to the examples in a short time, and is conducive to the construction of the SMO algorithm.

**Figure 3:**
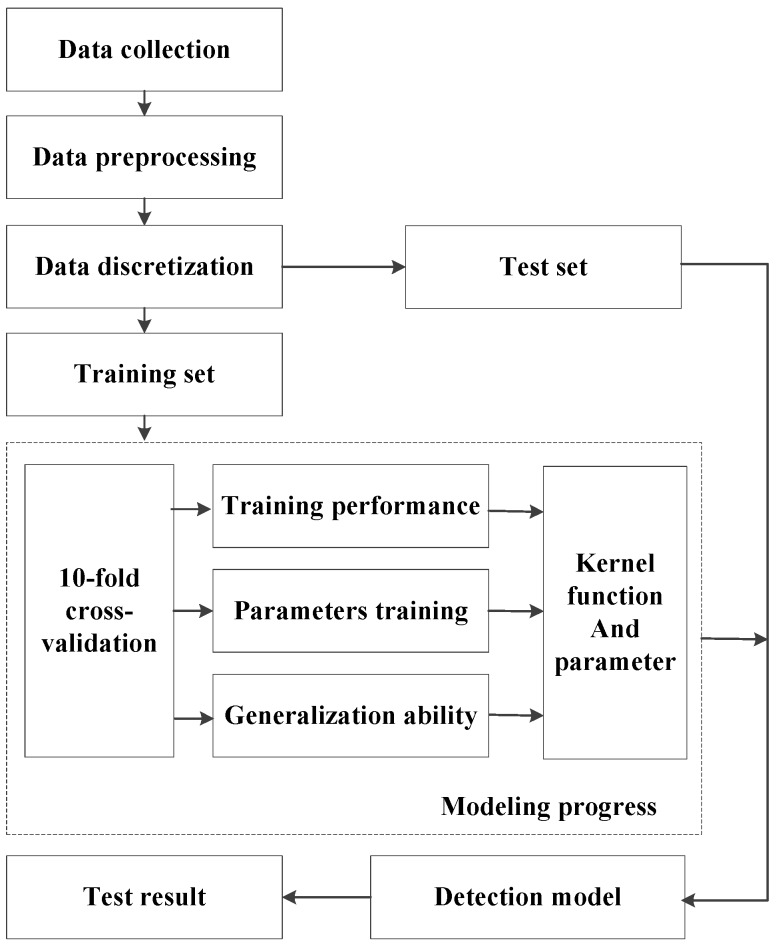
The SMO modeling process.

The advantage of this algorithm is that it is able to fill the missing value attributes of samples automatically; meanwhile, multiple classification problems are solved by using pairs’ classification in this model [39]. The entire inner iteration is avoided as two Lagrange multipliers can be used if the numerical QP problems exist. In addition, the training speed is improved and does not require extra matrix storage by SMO algorithm compared with SVM. The modeling progress of SMO algorithm is shown in Figure 3.

The 10-fold cross validation and grid method is employed to optimize both the parameters and the kernel function, which is suitable for developing this classification model, and the training result shows that the model presented has highest accuracy when the value of *c* (the punishment factor) is 1 and the value of *γ* (the tolerance parameter) is 0.001; the PLOY (polynomial kernel function) is selected as the kernel function of SMO and is described as follows:

(4) $$k(x,x_{i})={\left((x*x_{i})+1\right)}^{d}$$

where *x* is the expected type of traffic event; *x_i_* is the sample of the training set; *d* is the kernel function parameter; the value of *d* is 1.0 in this study.

### 2.3. Evaluation Criteria for Detection Model

After filtering most of the features using the MB-SMO algorithm, it is necessary to select a suitable method to evaluate the accuracy of the identification. For this purpose, a wrapper method based on the area under the ROC curve (AUC) is employed in this study. The ROC curve is the integrated index that reflects the sensitivity and specificity of continuous variables. By setting out a number of different thresholds of continuous variables, the ROC calculates a series of sensitivity and specificity, using sensitivity as the true positive rate (TPR), false positive rate (FPR), Recall (R), and F-measure (see Equations (5)–(9)) described as follows. A higher AUC implies that the classification algorithm can achieve a better performance [40]:

(5) $$\text{TPR}=\frac{TP}{TP+FN}$$

where *TP* represents the number of events correctly classified as belonging to class A, and *FN* represents the number of events incorrectly classified as not belonging to class A. TPR could represent the percentage of members of class A correctly classified as belonging to class A:

(6) $$\text{FPR}=\frac{FP}{FP+TN}$$

where *FP* represents the number of events incorrectly classified as belonging to class A, and *TN* represents the number of events correctly classified as not belonging to class A. FPR could represent the percentage of members of class A incorrectly classified as belonging to class A:

(7) $$P=\frac{TP}{FP+TP}$$

(8) $$R=\frac{TP}{FP+TN}$$

(9) $$F-\text{measure}=\frac{2*PR}{P+R}$$

where *P* represents the precision of the true positive event; *R* is called Recall and indicates the precision of the result in classifying all the events.

## 3. Experiment Study and Results

### 3.1. Equipment and Experiment Process

#### 3.1.1. Participants

Twenty-two participants, including 18 males and four females, were recruited to complete the tests. As a rule, related studies reported that males are involved in more crashes and easily receive more traffic violations than females [41]. Thus, more male drivers were employed in this study. The ages of participants were between 20 and 60 (with the mean = 42.27 years, SDV = 9.07); all of the participants were licensed drivers with at least three years of experience (with mean = 13.5, SDV = 6.06) and frequent drivers (i.e., they drove at least three or more days a week). Drivers were paid $50 per experiment, which lasted approximately 2 h.

#### 3.1.2. Experiment Instrument

**Figure 4:**
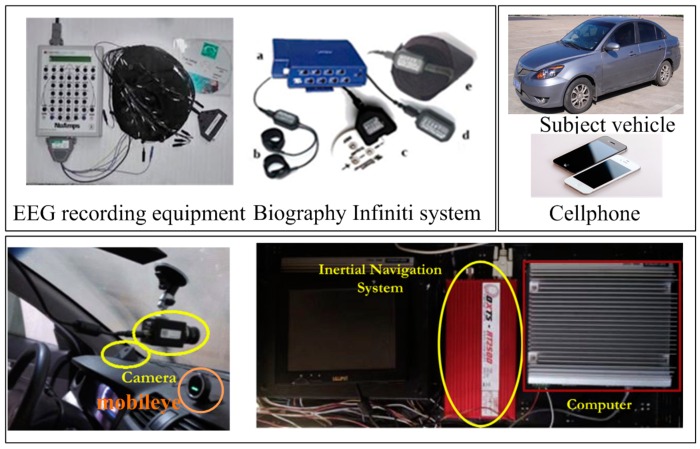
Installation of the data collection system.

To investigate the drivers’ characteristics and vehicle’s motion features in different traffic scenarios, a data collection system was developed and installed on a subject vehicle, as shown in Figure 4. It was equipped with many instruments and sensors, including cameras, industrial computers, an inertial navigation system, EEG recording equipment, Biography Infiniti System, and a cellphone (for acquiring the acceleration of vehicle). The data collection system could collect three types of signals, and the sample rate of each variable is shown in Table 1, including the signals related to the driver, the signals related to the vehicle, and the signals related to the road and environment. The signals related to the driver included blood volume plus (BVP), skin conductance (SC), respiration rate (RR), and PERCLOS, which was used to evaluate a driver’s physiological state during the field experiments. The evaluation of the vehicle’s states always relied on the signals related to the vehicle, which included speed, brake pressure, steering wheel angle and so on. Moreover, the information data (collected by the Mobileye and cellphone) was used to identify the relationship between the external environment and the subject vehicle.

#### 3.1.3. Driving Protocol

**Figure 5:**
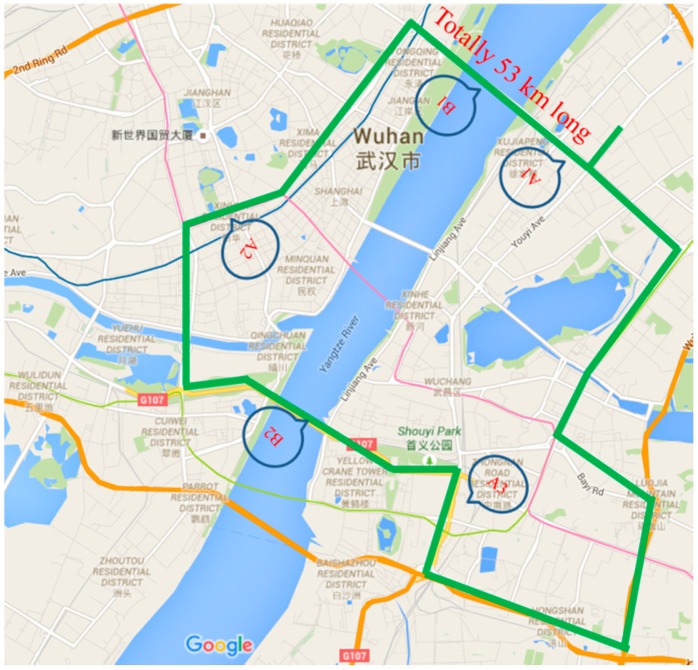
The test route.

A 53 km long ring road (shown in Figure 5) in Wuhan, China was selected as the test route to conduct the experiment. It includes 45 traffic lights, three large-scale business districts (A1, A2, and A3 in Figure 5), two bridges (B1 and B2 in Figure 5), many other normal traffic facilities, and the speed limit is 60 km/h. The traffic flow was busy, thus, long traffic delays easily occur and the possibility of hazardous traffic events occurring is increased. Moreover, considering the driver's safety in the field experiment, the weather conditions were pretty good with good visibility, and the road was dry enough with a high friction coefficient. The drivers were asked to fill out a survey which includes the drivers’ individual characteristics information (such as the participants’ age, sex, driving experience and so on). Then, they started the driving test and self-reported the risk levels when encountering traffic events. Each driver was asked to finish the driving test twice. The drivers were instructed to drive as normally as possible. To avoid traffic violations and collect the experimental data, two other assistants were recruited to stay on-board during the whole test. One of the assistants was an experienced driver (i.e., the assistant was a taxi driver or a driving coach), who was asked to record the participants’ self-reporting, assess the risk level of the current level, and observe potential safety hazards. Another assistant operated the equipment expertly and copied the experiment data after each test.

### 3.2. Statistical Data Analysis

#### 3.2.1. Data Preprocessing

**Figure 6:**
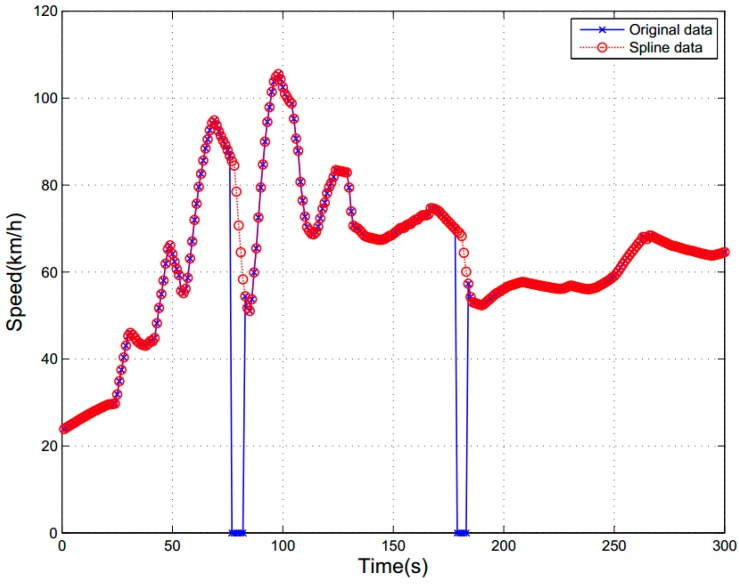
A sample of cubic spline interpolation.

Raw data (collected by different instruments) was processed in MATLAB and stored as a .mat file. It contained 27 features (including 20 raw features and seven statistical features, as shown in Table 1). Because of the instability and complexity of the data collection system, the emergence of outlying data points was inevitable. To ensure the accuracy and reliability of data, a method of cubic spline interpolation, which had already been proven as a suitable way to restore the invalid data, time-related errors, speed mutation data of driving in previous study [42], was employed. Using the vehicle’s speed as an example, as shown in Figure 6, the blue points represent the original speed data which were selected from the raw data (the collection time is 5 min long, the data points from 77 to 82 s and from 179 to 183 s are abnormal or display errors). The red points are the speed data, which were fitted by using the cubic spline interpolation algorithm. From Figure 6, we can see that this interpolation method restores the reduction of data points successfully.

#### 3.2.2. The Level of Traffic Events

**Figure 7:**
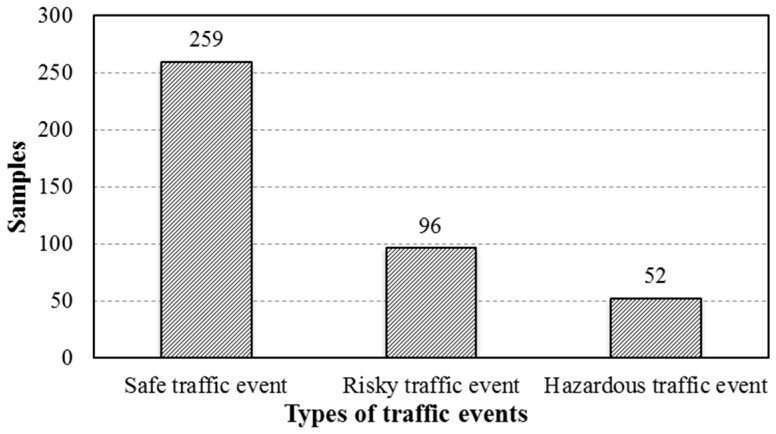
The number of different types of traffic events.

The record of traffic events which were assessed by the driver, the assistant, and the expert is shown in Table 2. The traffic events were divided into three types (hazardous traffic events, risky traffic events, and safe traffic events) as defined in the introduction. During the field test, the driver and assistant were asked to give an evaluation when traffic events occurred. If the self-report from the driver was similar to that of the assistant, the traffic event would be considered as the type which they reported. Otherwise, the expert (from the transportation management department) would be asked to make a further judgment based on the recorded video. Finally, 407 samples of traffic events (shown in Figure 7) were selected after the judgment.

### 3.3. The Result of Feature Selection

**Figure 8:**
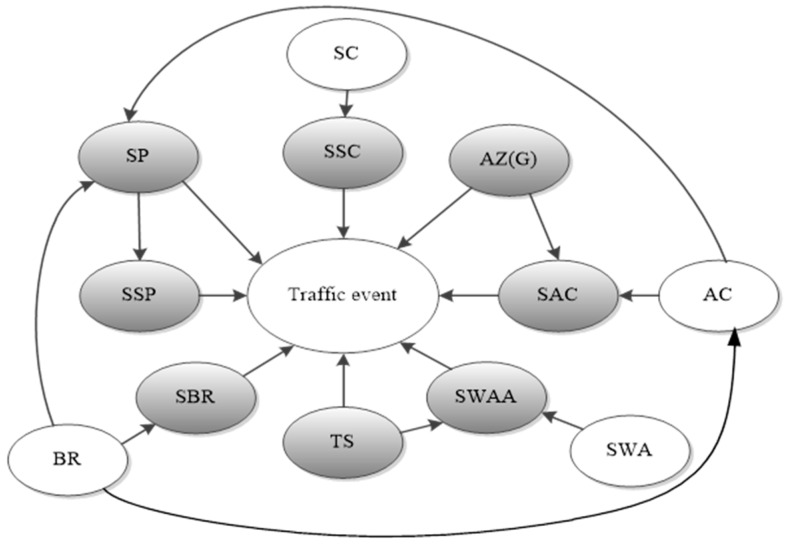
The feature selection using the MB algorithm.

In order to analyze the relationship between the impact factors and different types of traffic events, the unsupervised discretization method called the Numeric to Nominal method (which could be conducted directly by using the Weka software) was employed to discretize continuous features before feature selection. Then, the algorithm of MB was used to select the key factors, which are shown in Figure 8, the grey features are the objective features which related to a traffic event; eight features (including SP, SSC, SSP, SBR, TS, SWAA, SAC, AZ (G)) were selected to predict the risk level of a traffic event. The relationship between the selected features and other variables is also presented in this figure.

**Figure 9:**
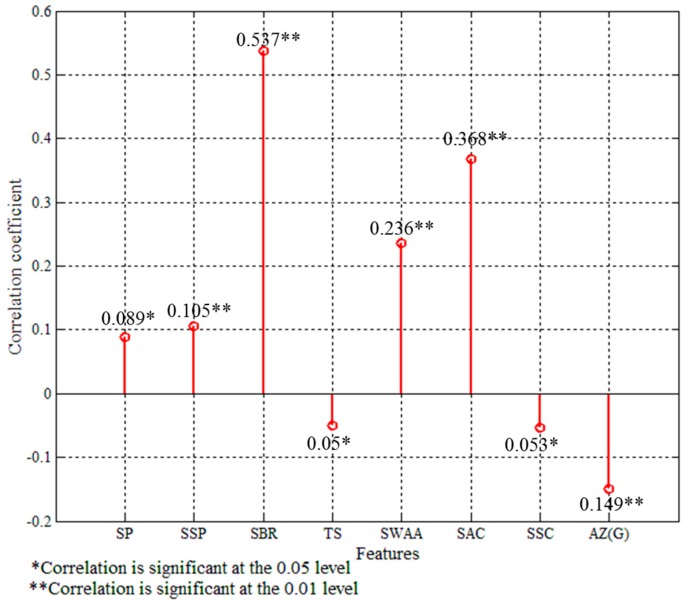
The correlations between eight selected features and traffic events.

In order to examine the correlations between the selected features and different types of traffic events, the Pearson correlation coefficient test was adopted. As shown in Figure 9, the Pearson correlation coefficient test presents that vehicle’s speed (correlation coefficient = 0.089, *p* = 0.046 < 0.05), standard deviation of the vehicle’s speed (correlation coefficient = 0.105, *p* = 0.000 < 0.05), standard deviation of brake pressure (correlation coefficient = 0.537, *p* = 0.000 < 0.05), turn signal (correlation coefficient = −0.05, *p* = 0.049 < 0.05), steering acceleration (correlation coefficient = 0.236, *p* = 0.000 < 0.05), standard deviation of acceleration (correlation coefficient = 0.0368, *p* = 0.000 < 0.05), standard deviation of skin conductance (correlation coefficient = −0.053, *p* = 0.047 < 0.05), and acceleration Z(G) (correlation coefficient = −0.149, *p* = 0.000 < 0.05) are significant correlated to the different traffic events, using the significance level of alpha = 0.05 respectively. Moreover, the correlation test also indicates that the relationship between SP, SSP, SWAA, SBR, SAC and traffic event is significant positive correlation. A negative correlation is existed between TS, SSC, AZ(G) and different types of traffic events.

The partial correlation test was employed to analyze the correlations among eight selected features while assumed the traffic event as the control variable. The results in Table 3 shows that turn signal, standard deviation of skin conductance, acceleration Z(G) are no significant correlated to other features, with the correlation is significant at the level of alpha = 0.01. Although the relationship among other four variables (SP, SSP, SBR, and SAC) is correlated, it is reasonable that all of these four features influence traffic safety directly.

### 3.4. The Traffic Event Detection Model

In this section, we empirically evaluate the performance of the SMO algorithm with the polynomial kernel function (PLOY) by comparing it with other commonly used kernel functions, such as the normalized polynomial kernel function (NOR), precomputed kernel function (PUK), and radial basis function (RBF). In order to evaluate the effectiveness of the proposed method of MB, the most representative selection methods (PCA and DT) are considered as comparisons. Finally, to evaluate the performance of the identification model, the algorithms of ID3, NB, FTA, and RBFNETWORK (which were introduced in the Introduction) are used to compare with the proposed SMO algorithm. All of the algorithms are implemented in Java and applied in the Weka platform, and the data analysis experiment is conducted using a 64-bit Windows computer with 2.4 GHz CPU, 8 GB RAM.

#### 3.4.1. Results of Different Kernel Functions

**Figure 10:**
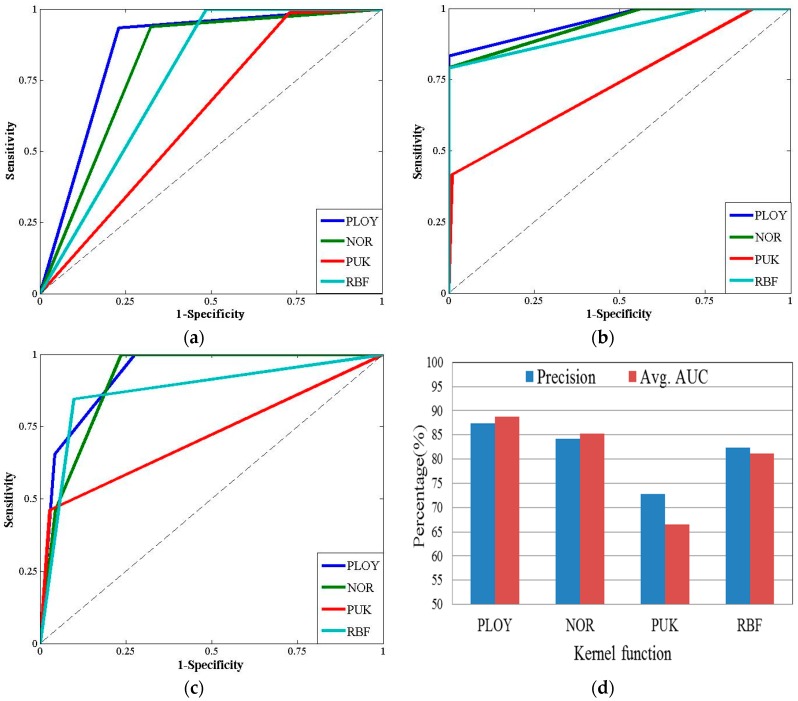
Results of different kernel functions. (**a**) The ROC curve of safe traffic events; (**b**) The ROC curve of risky traffic events; (**c**) The ROC curve of hazardous traffic events; (**d**) The AUC and precision.

A comparison between SMO with the polynomial kernel function and three other kernel functions with respect to the ROC curve and accuracy is shown in Figure 10. The 10-fold cross validation method was employed to evaluate the accuracy of different algorithms. The results shown in this figure clearly present that SMO with PLOY is superior to SMO with NOR, PUK, and RBF with respect to AUC and precision (see Figure 10d). Moreover, the ROC curve shows that SMO with the polynomial kernel function is the most effective one (the AUC reached 88.88% and the accuracy was 87.46%) when identifying the different types of traffic events (see Figure 10a–c).

#### 3.4.2. Results of Different Feature Selection Algorithms

To evaluate the performance of the Markov Blanket, the SMO algorithm using the polynomial kernel function was employed to classify the different types of traffic events. The results in Table 4 show the performance of the SMO classifier using three feature selection algorithms over the 10-fold cross validation and grid method. The result indicates that the number of selected features of MB is the smallest compared with the other algorithms. Moreover, from Table 4, we can also see that the accuracy of classification of SMO-MB (which reached 87.5%) is the highest in four different types of feature selection method with SMO. The results of the average of TPR, FPR, and AUC also indicate that the SMO-MB selected fewer features to obtain a better classification.

#### 3.4.3. Results of Different Classifiers

The results presented in Table 5 show the performances of six different classifiers using the Markov Blanket feature selection algorithm; the 10-fold cross validations method was employed to verify the accuracy of six classification algorithms. As shown in Table 5, the MB-SMO achieved better accuracies compared with the other five algorithms with respect to the average true positive rate (0.875), false positive rate (0.153), and accuracy (0.875), which verifies that the MB-SMO is superior to the other five classifiers. In addition, although the area under the ROC curve in MB-SMO is not the biggest among the six algorithms, it did reach 0.888, which indicates that the MB-SMO algorithm was effective enough to be used to identify the different types of traffic events.

**Figure 11:**
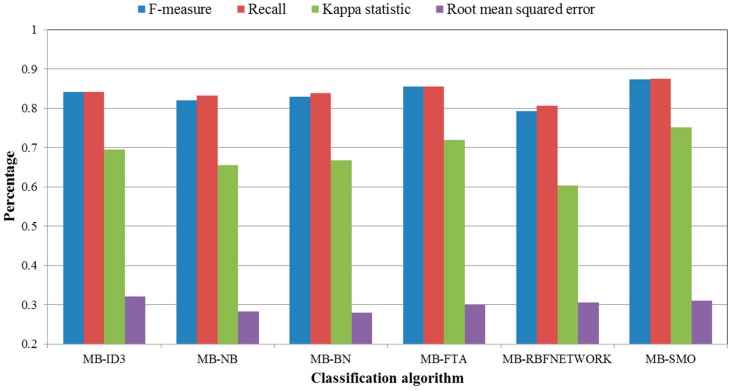
The statistical analysis of the classification.

Four indexes (including F-measure, Recall, Kappa statistic, and Root mean squared error) were chosen to evaluate the performance and effectiveness of the classification. As shown in Figure 11, F-measure and Recall in this experiment show that MB-SMO (0.874 and 0.875, respectively) outperforms the other methods. The kappa statistic of six algorithms (MB-SMO > MB-FTA > MB-ID3 > MB-BN > MB-NB > MB-RBFNEWORK) indicates that the consistency between the prediction value and the actual value is the strongest when using MB-SMO compared with the other five algorithms. The root mean squared error has no significant difference among the six algorithms.

## 4. Discussions and Conclusions

A real-life driving data were collected through a field driving experiment. The data continuously collected from the driver, vehicle, environment, and road provided the opportunity to evaluate not only the traffic accident risk, but also hazardous traffic events, which easily result in a crash. This study tried to obtain the risk factors associated with the occurrence of hazardous traffic events using real vehicle experiment data; these experiments were conducted in the mixed traffic of a Chinese metropolitan area, i.e., Wuhan, and the subject vehicle was developed by the ITS center of Wuhan University of Technology (WUT).

The statistical analysis of the original data indicated that hazardous traffic events rarely occurred in the environment of urban road driving; they were only reported 52 times in 44 groups of driving experiments; representing only 12.7% of the total selected traffic events. However, they had a substantial impact on traffic safety. Many studies have presented that drivers, especially inexperienced drivers, always tend to become nervous and commit operating errors in hazardous scenarios [43]. Thus, it is necessary to find a way to identify hazardous events effectively so warning can be given to drivers or the operational power can be automatically taken from the driver by an intelligent vehicle.

In the field of driving safety assistance, considering the reasons of economics and technology, most of the previous studies focused only on one particular type of hazardous or risky traffic events. However, the common characteristics of hazardous traffic events have not been analyzed. Dangerous scenes are always tracked by using visual sensors data or vehicle motion data. In this study, we try to extract a set of common features with respect of humans, vehicles, and other features related to the environment. The human factors, such as a driver’s blood volume plus, skin conductance, respiration rate, and PERCLOS, are collected by using the biological sensors and EEG recording equipment. The other two types of data are collected by using a vehicle’s CAN and many dedicated devices.

To extract the main factors that have significant impacts on hazardous or risky traffic event during the driving process, a feature selection algorithm called Markov Blanket was used. Eight factors (including speed, the standard deviation of speed, the standard deviation of SC, the standard deviation of brake pressure, turn signal, the acceleration of steering, the standard deviation of acceleration, and acceleration in Z (G)) were selected from all 27 features by MB. The results of Pearson correlation coefficient test indicate that these eight selected factors are significant correlated to the traffic events. It is well known that the indexes of speed, the standard deviation of speed, the standard deviation of brake pressure, and the standard deviation of acceleration are contribute to the longitudinal control of a vehicle. The acceleration in Z (G) could express the road alignment where the vehicle located, as an example, the position where vehicle located in is an uphill road while the value of acceleration in Z (G)) is positive. The SSC could express a driver’s physiological state during the driving process. Moreover, the indexes of turn signal and the acceleration of steering could indicate a driver’s lane change intention or behavior. All in all, these eight factors present a driver’s behavior with respect to lateral control, longitudinal control, and current physiological state. Thus, it is reasonable to select these factors to evaluate the hazardous scenarios.

**Figure 12:**
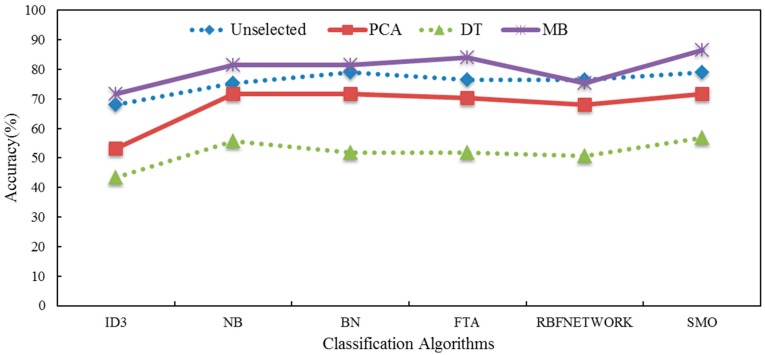
The evaluation of the classification algorithms.

**Figure 13:**
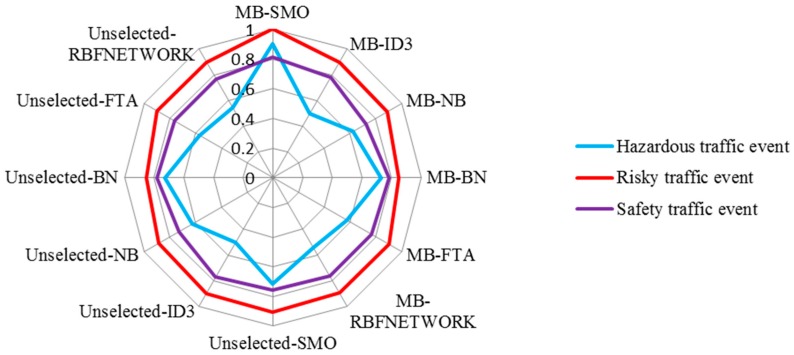
The radar map of the three traffic event styles.

A comprehensive evaluation of different algorithms to identify the traffic event styles is shown in Figure 12, which plots the average recognition accuracy of the different classification algorithms. Eighty new samples (the data was collected by others five on-road experiments and the participants were recruited from the 22 volunteers who had already attended the experiment before) were chosen to verify whether the MB-SMO is the most appropriate algorithm for evaluating the traffic event styles. The result in Figure 12 showed that the MB-SMO is ranked best when compared with other classification algorithms. Meanwhile, all the classification algorithms presented significant improvement after using the Markov Blanket feature selection method with respect to the accuracy of prediction. However, two feature selection algorithms could not achieve similar effectiveness. In addition, the radar map (see Figure 13) presented the precision of three traffic event styles when using the different classification algorithms. There were significant differences among the three traffic event styles tested. MB-SMO receives the highest scores for all three traffic event styles. Meanwhile, the identification accuracy was significantly increased by using MB-SMO compared to using Unselected-SMO. All 96 groups of risky traffic events are identified correctly and the prediction accuracy of hazardous traffic event and safe traffic event reached 90% and 81.1% respectively, by the algorithm of SMO-MB. However, the prediction accuracy of each type of traffic event in algorithms of ID3, NB, BN, FTA, and RBF using the features selection method of MB couldn’t present significant increasing compared to using the full features. All in all, the results in Figure 12 and Figure 13 indicate that the use of the Markov blanket can help us filter out the useless or irrelevant features so that it can improve the prediction accuracy. Meanwhile, the SMO algorithm has proved that it is a suitable method employed to identify different types of traffic events.

The overall conclusion is that the MB-SMO is the most appropriate one, compared to the other identification algorithms with different feature selection methods. Although the sample size of this study is limited, a significant relationship between traffic event styles and risk factors, such as the characteristics of lateral control, longitudinal control, and physiological state, was observed. More importantly, a traffic event styles classification model was built and the results demonstrate that the classification model is effective and reliable when evaluating the hazardous traffic events.

Nevertheless, due to the relatively small, voluntary sample size (consisting of only 22 participants), this study ignored the relationship between driver’s personality and the occurrence of traffic events. However, many scholars have put forward that a driver’s sex, experience and age also have impact on the occurrence of the hazardous traffic events [43]. It is suggested that a larger group of experiment should be carried out in the future to enrich the types of factors. Note that, this experiment was only conducted using a set of special routes of an urban road in Wuhan, China (one of the cities in which traffic is always congested and complex); a similar conclusion should be proven under other traffic conditions, such as a freeway, another city, and so on. In addition, the relationship between road conditions, environment (such as weather condition) and hazardous traffic event also should be considering about in the future study. Finally, our database does not include crashes due to the safety of the driving experiment, the response variable for traffic events was only summarized for three types (hazardous traffic events, risky traffic events, and safe traffic events), and the risk factors related to hazardous traffic events may be different from eight factors we selected; our model was not able to consider them because these data were not included in our database.

## Figures and Tables

**Table 1:** The characteristics of the collected data.

Variable	Equipment	Sampling Rate	Tag
Related to driver			
BVP	Biography Infiniti system	256 Hz	BVP
STD of BVP			SBVP
SC	Biography Infiniti system	256 Hz	SC
STD of SC			SSC
RR	Biography Infiniti system	256 Hz	RR
STD of RR			SRR
PERCLOS	EEG recording equipment	1000 Hz	POS
Related to vehicle			
Speed	CAN in Vehicle	25 Hz	SP
STD of speed			SSP
Brake	CAN in Vehicle	25 Hz	BR
STD of brake			SBR
Turn signal	CAN in Vehicle	25 Hz	TS
Course angle	CAN in Vehicle	25 Hz	CS
Pitching angle	CAN in Vehicle		PA
Steering wheel angle	Steering angle sensor	30 Hz	SWA
STD of steering			SSWA
Steering acceleration	Steering angle sensors	30 Hz	SWAA
STD of steering acceleration			
Acceleration	Inertial Navigation system	100 Hz	AC
STD of acceleration			SAC
Related to road and environment			
The spacing to left lane line	MobileyeC2-270	15 Hz	SLL
The spacing to right lane line	MobileyeC2-270	15 Hz	SRL
Time headway	MobileyeC2-270	15 Hz	TH
Lane departure	MobileyeC2-270	15 Hz	LD
Acceleration X(G)	Cellphone	256 Hz	AX(G)
Acceleration Y(G)	Cellphone	256 Hz	AY(G)
Acceleration Z(G)	Cellphone	256 Hz	AZ(G)

**Table 2:** A sample of the traffic event record.

Time	Traffic Event		
	Self-Report	Assistant Report	Expert Record
14 October 2014; 9:00; 12	0	0	0
14 October 2014; 9:08; 11	2	2	2
14 October 2014; 9:25; 15	1	0	1
14 October 2014; 9:28; 16	0	1	1

Note: a score of 0 indicates a safe traffic event; a score of 1 indicates a risky traffic event; a score 2 indicates a hazardous traffic event.

**Table 3:** The results of partial correlation test while control the variable of traffic event.

Control Variables			SP	SSP	SBR	TS	SWAA	SAC	SSC	AZ(G)
Traffic event	SP	Correlation	1.000	0.391	−0.179	0.030	0.027	0.246	−0.014	−0.069
		Sig.	0.000	0.00	0.040	0.540	0.593	0.002	0.772	0.163
	SSP	Correlation		1.000	0.169	0.015	0.105	0.007	−0.025	−0.147
		Sig.		0.000	0.002	0.340	0.034	0.892	0.013	0.617
	SBR	Correlation			1.00	−0.18	0.01	−0.037	0.058	0.054
		Sig.			0.000	0.721	0.845	0.461	00.242	0.276
	TS	Correlation				1.00	−0.114	0.056	0.000	−0.016
		Sig.				0.000	0.021	0.263	0.0993	0.742
	SWAA	Correlation					1.00	−0.002	−0.009	0.034
		Sig.					0.000	0.975	0.852	0.493
	SAC	Correlation						1.00	0.015	0.031
		Sig.						0.000	0.764	0.529
	SSC	Correlation							1.00	−0.014
		Sig.							0.000	0.777
	AZ(G)	Correlation								1.000
		Sig.								0.000

Notes: Sig. is significance (2-tailed), and correlation is significant at the 0.01 level.

**Table 4:** Results of different feature selection algorithms using SMO.

Algorithms	Features	Avg. TPR	Avg. FPR	AUC	Accuracy
SMO-unselected	27	0.826	0.184	0.85	0.825
SMO-PCA	11	0.757	0.365	0.718	0.757
SMO-DT	9	0.595	0.57	0.51	0.595
SMO-MB	**8**	**0.875**	**0.153**	**0.888**	**0.875**

Avg. means Average.

**Table 5:** Results of different classifiers with MB feature selection.

Algorithms	Avg. TPR	Avg. FPR	AUC	Accuracy
MB-ID3	0.842	0.156	0.81	0.744
MB-NB	0.833	0.227	0.912	0.833
MB-BN	0.838	0.214	0.915	0.838
MB-FTA	0.855	0.154	0.893	0.855
MB-RBFNETWORK	0.806	0.244	0.893	0.806
MB-SMO	**0.875**	**0.153**	0.888	**0.875**
